# PROTOCOL: New‐Onset Diabetes Mellitus Post COVID‐19 Infection: A Protocol for Systematic Review and Meta‐Analysis: A Systematic Review

**DOI:** 10.1002/cl2.70069

**Published:** 2025-09-30

**Authors:** Emma Cocking, Joseph Daher, Majid Alabbood

**Affiliations:** ^1^ School of Medicine The University of Notre Dame Australia Sydney New South Wales Australia; ^2^ Department of Medicine Gold Coast University Hospital Southport Queensland Australia

## Abstract

**Background:**

Emerging evidence suggests that coronavirus disease 2019 (COVID‐19) infection may cause diabetes mellitus in patients without a prior history of the condition.

**Objective:**

This review aims to determine the incidence of new‐onset diabetes mellitus in COVID‐19 patients compared to individuals without COVID‐19, including rates of diabetic ketoacidosis, hyperglycaemia, mortality, and intensive care unit admission. Subgroup analyses will investigate patients receiving corticosteroid therapy for COVID‐19, patients admitted to hospital due to COVID‐19, and the incidence of new‐onset diabetes mellitus in relation to diabetes mellitus worldwide. The incidence of new‐onset diabetes mellitus post‐infection after a 6–12‐month follow‐up will also be reported.

**Methods:**

This protocol follows the Preferred Reporting Items for Systematic Review and Meta‐Analysis Protocols (PRISMA‐P) guidelines and is registered in PROSPERO (CRD42023457569). Eligible studies will include published and peer‐reviewed cohort studies in English, after 1 December 2019. PubMed, Medline, Embase, and Scopus will be systematically searched. Included studies should compare new‐onset diabetes mellitus incidence in COVID‐19 patients against a control group without COVID‐19. Two independent reviewers will extract data from included studies and assess risk of bias using the Newcastle‐Ottawa quality assessment scale. A random‐effects meta‐analysis will compare the relative risk of new‐onset diabetes mellitus post COVID‐19 infection compared to non‐infected individuals.

**Conclusion:**

The findings of this review will contribute to understanding the bidirectional relationship between COVID‐19 and diabetes mellitus and inform clinical management strategies for patients at risk.

**Systematic Review Registration:**

PROSPERO CRD42023457569.

## 1 Background

Severe acute respiratory syndrome coronavirus 2 (SARS‐CoV‐2) is the source of the global pandemic known as coronavirus disease 2019 (COVID‐19), resulting in nearly 7 million deaths worldwide (World Health Organization 2023). While the most common symptoms of COVID‐19 include fever, cough and dyspnoea, recent evidence suggests that COVID‐19 infection can also lead to new‐onset diabetes mellitus (NODM) (Aslam 2023; Choi et al. 2023; Kim et al. 2023; Rubino 2022). For the purpose of this review, NODM will be defined as any diagnosis of hyperglycaemia meeting the International Diabetes Federation (IDF) criteria for diabetes mellitus (DM) (International Diabetes Federation 2021) in individuals with no prior history of the condition. NODM post‐COVID‐19 infection commonly presents as type 2 DM driven by insulin resistance; however, cases of type 1 DM, driven by autoimmune β‐cell destruction, following COVID‐19 infection have also been reported (Kim et al. 2023; Stathi et al. 2023).

A bidirectional relationship between COVID‐19 and DM has been observed in the last few years (Cao et al. 2023; Lima‐Martínez et al. 2021). It is well documented that DM is a significant risk factor for poor prognosis of COVID‐19 (Landstra and de Koning 2021). COVID‐19 patients with established DM have a greater likelihood of intensive care unit (ICU) admission, mechanical ventilation and death (Seiglie et al. 2020). Evidence has also emerged that COVID‐19 can cause direct damage to the endocrine function of the pancreas, leading to uncontrolled hyperglycaemia and the development of NODM in patients with no prior history of the condition (Geetha et al. 2023; Müller et al. 2021; Singh and Khunti 2022). Systemic corticosteroid therapy is often used in the treatment of severe COVID‐19 infection. It is well documented that corticosteroid use is associated with steroid‐induced hyperglycaemia and an increased risk of NODM, particularly in hospitalised patients (Cho and Suh 2024; Fetters et al. 2022).

Previous systematic reviews have explored the association of incident diabetes within 1–6 months post COVID‐19 infection (Harding et al. 2023; Ssentongo et al. 2022; Zhang et al. 2022). However, this systematic review and meta‐analysis will extend on past research by performing subgroup analyses based on systemic corticosteroid therapy, severity of COVID‐19 infection, type of NODM (type 1, type 2), age, and 6–12‐month follow‐up.

## 2 Objectives

The primary objective of this systematic review and meta‐analysis is to determine the incidence of NODM in patients within 1 month following COVID‐19 infection compared to people without COVID‐19. Rates of diabetic ketoacidosis (DKA) and hyperglycaemia will be compared between the two groups, as well as secondary outcomes of mortality rate and rates of ICU admission.

The review also aims to investigate the incidence of NODM following COVID‐19 infection in the following subgroups: patients who underwent systemic corticosteroid therapy for COVID‐19, patients who were admitted to hospital due to COVID‐19, and the incidence of NODM in relation to the prevalence of DM worldwide. This review will also report the incidence of NODM post‐infection after a 6–12‐month follow‐up to assess whether patients still meet the criteria for diabetes.

## 3 Methods

This protocol was written in accordance with the Preferred Reporting Items for Systematic Review and Meta‐Analysis Protocols (PRISMA‐P) 2015 statement (Moher et al. 2015). This protocol is registered in the PROSPERO International Prospective Register of Systematic Reviews (registered 01/09/2023: CRD42023457569).

### 3.1 Eligibility Criteria

The PICO (patient, intervention/exposure, comparison, outcome) method was used to determine the eligibility criteria and search strategy for this systematic review and meta‐analysis (Eriksen and Frandsen 2018).

#### 3.1.1 Types of Studies

Published and peer‐reviewed, prospective and retrospective cohort studies will be included in the review. Case reports, case series, letters, commentaries, reviews and clinical trials will be excluded. Studies will be eligible if they are published in English or have an English‐language version available and were published after the 1st December 2019.

#### 3.1.2 Participants

Studies that include adult and paediatric patients may be included. Studies in which patients received corticosteroids may also be included. COVID‐19 vaccination status will not impact participants' eligibility. Patients with existing comorbidities may be considered for inclusion; however, patients with an existing diagnosis of DM will be excluded.

#### 3.1.3 Exposure

The exposure of interest is a COVID‐19 diagnosis. For a study to be eligible, patients must have been clinically diagnosed with a current COVID‐19 infection by either a reverse transcription polymerase chain reaction (RT‐PCR) or a rapid antigen test.

#### 3.1.4 Comparator

Included studies should compare the incidence of NODM in patients following COVID‐19 infection against a control group without a diagnosis of COVID‐19. The comparator can be population controls or hospitalised patients without COVID‐19. Participants in the control group should not have been diagnosed with COVID‐19 in the previous 30 days.

#### 3.1.5 Types of Outcome Assessments

The primary outcome that will be assessed is the incidence of NODM type 1 or type 2, as well as DKA and hyperglycaemia in patients post COVID‐19 infection. A diagnosis of diabetes will be defined according to the IDF criteria (Table 1) (International Diabetes Federation 2021). Secondary outcomes of mortality rate and rates of ICU admission will also be included.

**Table 1:** International Diabetes Federation (IDF) criteria for diagnosis of diabetes mellitus.

Diagnostic test	Criteria
Fasting plasma glucose	≥ 7.0 mmol/L
2‐h plasma glucose during an oral glucose tolerance test	≥ 11.1 mmol/L
Random plasma glucose	≥ 11.1 mmol/L
HbA1c	≥ 6.5%

*Note:* Patient must meet at least one of the criteria (International Diabetes Federation 2021).

### 3.2 Search Methods

The electronic databases PubMed (Medline), Medline (Ovid), Embase (Elsevier) and Scopus (Elsevier) will be systematically searched. EndNote will be used to manage all the study records. Resources from a librarian were used to guide the development of the search strategy.

The PubMed Medical Subject Headings (MeSH) database will be used to identify search terms relating to COVID‐19, DM, new‐onset and cohort studies (Table 2). An example search strategy is included in the supporting materials (Table S1). These keywords will be used to conduct a thorough literature search. To ensure completeness, ancestral citation searching will be performed by checking the reference lists of eligible studies and previously published systematic reviews. In addition, forward citation searching will also be conducted.

**Table 2:** Search strategy.

	Concept	Keywords
1	COVID‐19	(‘COVID‐19’ OR COVID OR COVID19 OR ‘SARS‐CoV‐2’ OR ‘2019 Novel Coronavirus Disease’ OR ‘2019 Novel Coronavirus Infection’ OR ‘Severe Acute Respiratory Syndrome Coronavirus 2’ OR ‘SARS Coronavirus 2 Infection’ OR ‘2019‐nCoV Disease’ OR ‘Disease, 2019‐nCoV’ OR ‘RT‐PCR’ OR ‘Antigen test’)
2	Diabetes mellitus	AND (‘Diabetes mellitus’ OR Diabet* OR Hyperglycaem* OR Hyperglycem*)
3	New onset	AND (‘New‐onset’ OR ‘New onset’ OR ‘Newly diagnosed’ OR ‘New diagnosis’)
4	Cohort study	AND (Cohort OR Longitudinal OR ‘Follow‐up’ OR Prospective OR Retrospective)

*Note:* ‘*’ – Truncating symbol.

### 3.3 Data Collection and Analysis

#### 3.3.1 Study Selection Process

Two independent reviewers will initially screen the titles and abstracts of studies and then will further perform a full‐text screen according to eligibility criteria. Disagreement will be resolved through discussion between the two reviewers, and further disagreement will be settled through consultation with a third investigator. Duplicated results will be excluded, and where there are studies using overlapping cohorts, only the study with the larger sample size will be included. If it is deemed necessary, additional information or data will be obtained by contacting the authors of the eligible studies. A PRISMA flowchart (Page et al. 2021) will be used to document the screening process and will be reported in the final systematic review.

#### 3.3.2 Quality Assessment

The risk of bias of the included studies will be assessed using the Newcastle‐Ottawa quality assessment scale for cohort studies (Wells et al. 2021), which rates cohort studies based on selection, comparability and outcome, with a maximum score of 9. Studies will be categorised as good, fair or poor quality according to the Agency for Health Research and Quality (AHRQ) criteria. The critical appraisal will be performed by two independent reviewers, and discrepancies will be discussed with a third reviewer. Results of the Newcastle‐Ottawa quality assessment will be presented in a summary table.

#### 3.3.3 Data Synthesis

The following data will be extracted from the included studies: year of publication, author names, country, study design, sample size, descriptive statistics (mean/median age in years, proportion female/male), proportion with COVID‐19, proportion with NODM, type of NODM (type 1, type 2) outcome assessment (mortality rates, rates of hospitalisation, and ICU admission), follow‐up time, corticosteroid treatment, and risk ratio. For studies where only counts are reported, we will estimate the risk ratio with a 95% confidence interval (Kirkwood and Sterne 2003). Where available, data on key moderating variables will be extracted, including ethnicity, pre‐existing conditions, vaccination status, and COVID‐19 variants. In the event of missing data, we will contact authors where possible. If unresolved, we will transparently report any missing data. A summary of findings table will be used to display the extracted data in the completed systematic review and meta‐analysis.

All statistical analyses will be performed using R software (version 4.1.1, Vienna, Austria) and will be calculated with 95% confidence intervals. *p*‐values < 0.05 will be considered statistically significant.

The incidence of NODM following COVID‐19 diagnosis will be calculated for each included study. A random‐effects model will be used to pool effect sizes and account for potential heterogeneity between studies (DerSimonian and Kacker 2007). The relative risk of NODM post COVID‐19 infection compared to the control population will be reported with 95% confidence intervals and 95% prediction intervals and will be represented visually using a forest plot.

Cochran's Q, *I* ^2^ and Tau^2^ statistics will be used to assess the heterogeneity between studies. I^2^ values of 25%, 50% and 75% will be interpreted as low, moderate, and high heterogeneity, respectively (Higgins et al. 2003).

Publication bias will be assessed using funnel plots and Egger's test (Egger et al. 1997).

Due to anticipated variability in reporting, formal moderator analyses may be limited. However, subgroup analysis will be performed on several variables including patients who underwent systemic corticosteroid therapy, diabetes type (type 1, type 2), age (adults ≥ 18 years old, children < 18 years old), severity of COVID‐19 infection (mild – outpatient, moderate – inpatient, severe – ICU/ventilated), and the incidence of NODM post COVID‐19 infection after a 6–12‐month follow‐up. Subgroup analysis will also be performed to compare between studies with healthy and unhealthy or hospitalised control groups, as well as by vaccination status. Extracted data will be used to calculate the risk ratio in each of these subgroups with 95% confidence intervals and prediction intervals to estimate the effect size. A random‐effects model will be used to account for potential heterogeneity between the studies (DerSimonian and Kacker 2007; Hunter and Schmidt 2000).

Finally, a sensitivity analysis will be performed by calculating the *E*‐value (Haneuse et al. 2019).

## 4 Conclusion

This systematic review and meta‐analysis will contribute to understanding the bidirectional relationship between COVID‐19 and DM and will provide insights that could inform clinical management strategies for at risk patients.

## Author Contributions

- Content: Emma Cocking, Joseph Daher, Majid Alabbood.
- Review methods: Emma Cocking, Joseph Daher, Majid Alabbood.
- Information retrieval: Emma Cocking, Joseph Daher.

## Conflicts of Interest

The authors declare no conflicts of interest.

## Preliminary Timeframe

The finalised submission of the systematic review manuscript will be within 18 months of the protocol approval.

## Plans for Updating This Review

This review will be updated every 2 years or as new relevant studies are published. The corresponding author will be responsible for monitoring the literature and incorporating updates as needed.

## Sources of Support

No funding was received for this study.

## Supporting information


**Supplementary TABLE S1:** Electronic search strategy for Embase (Elsevier).

## Data Availability

The data and supportive information will be made available upon completion of the systematic review and meta‐analysis.
