# Supplementary material for: PROTOCOL: New‐Onset Diabetes Mellitus Post COVID‐19 Infection: A Protocol for Systematic Review and Meta‐Analysis: A Systematic Review
Source: Campbell Syst Rev. 2025 Sep 30;21(4):e70069. doi: 10.1002/cl2.70069 (PMC12481428; doi:10.1002/cl2.70069)
Supplement: Supplementary file 1 — Supplementary TABLE S1: Electronic search strategy for Embase (Elsevier). [file CL2-21-e70069-s001.docx]

# Appendices

**Supplementary TABLE S1**: Electronic search strategy for Embase (Elsevier).

| **Database** | **Search terms** |
| --- | --- |
| Embase (Elsevier) | 1. ('covid-19'/exp OR 'covid-19' OR 'covid'/exp OR covid OR 'covid19'/exp OR covid19 OR 'sars-cov-2'/exp OR 'sars-cov-2' OR '2019 novel coronavirus disease'/exp OR '2019 novel coronavirus disease' OR '2019 novel coronavirus infection'/exp OR '2019 novel coronavirus infection' OR 'severe acute respiratory syndrome coronavirus 2'/exp OR 'severe acute respiratory syndrome coronavirus 2' OR 'sars coronavirus 2 infection'/exp OR 'sars coronavirus 2 infection' OR '2019-ncov disease'/exp OR '2019-ncov disease' OR 'disease, 2019-ncov' OR 'rt-pcr' OR 'antigen test'/exp OR 'antigen test') 2. ('diabetes mellitus'/exp OR 'diabetes mellitus' OR diabet* OR hyperglycaem* OR hyperglycem*) 3. ('new-onset' OR 'new onset' OR 'newly diagnosed' OR 'new diagnosis') 4. ('cohort'/exp OR cohort OR 'longitudinal'/exp OR longitudinal OR 'follow-up'/exp OR 'follow-up' OR prospective OR retrospective) 5. [01-12-2019]/sd NOT [13-12-3000]/sd   #1 AND #2 AND #3 AND #4 AND #5 |
